# Development of an algorithm to detect and reduce complexity of drug treatment and its technical realisation

**DOI:** 10.1186/s12911-020-01162-6

**Published:** 2020-07-08

**Authors:** Viktoria S. Wurmbach, Steffen J. Schmidt, Anette Lampert, Eduard Frick, Michael Metzner, Simone Bernard, Petra A. Thürmann, Stefan Wilm, Achim Mortsiefer, Attila Altiner, Lisa Sparenberg, Joachim Szecsenyi, Frank Peters-Klimm, Petra Kaufmann-Kolle, Walter E. Haefeli, Hanna M. Seidling

**Affiliations:** 1 Department of Clinical Pharmacology and Pharmacoepidemiology, University of Heidelberg, Im Neuenheimer Feld 410, 69120 Heidelberg, Germany; 2 Cooperation Unit Clinical Pharmacy, University of Heidelberg, Im Neuenheimer Feld 410, 69120 Heidelberg, Germany; 3 Chair of Clinical Pharmacology, Faculty of Health, University Witten/Herdecke, Alfred-Herrhausen-Straße 50, 58448 Witten, Germany; 4 Philipp Klee-Institute of Clinical Pharmacology, HELIOS University Clinic Wuppertal, Heusnerstraße 40, 42283 Wuppertal, Germany; 5 Institute of General Practice, Heinrich Heine University Düsseldorf, Moorenstraße 5, 40225 Düsseldorf, Germany; 6 Institute of General Practice, Rostock University Medical Center, Doberaner Str. 142, 18057 Rostock, Germany; 7 Department of General Practice and Health Services Research, Heidelberg University Hospital, Im Neuenheimer Feld 130.3, 69120 Heidelberg, Germany; 8 AQUA-Institute for Applied Quality Improvement and Research in Health Care, Maschmühlenweg 8–10, 37073 Göttingen, Germany

**Keywords:** Clinical decision support systems, Shared decision making, Polypharmacy, Self-administration, Medication regimen complexity

## Abstract

**Background:**

The increasing complexity of current drug therapies jeopardizes patient adherence. While individual needs to simplify a medication regimen vary from patient to patient, a straightforward approach to integrate the patients’ perspective into decision making for complexity reduction is still lacking. We therefore aimed to develop an electronic, algorithm-based tool that analyses complexity of drug treatment and supports the assessment and consideration of patient preferences and needs regarding the reduction of complexity of drug treatment.

**Methods:**

Complexity factors were selected based on literature and expert rating and specified for integration in the automated assessment. Subsequently, distinct key questions were phrased and allocated to each complexity factor to guide conversation with the patient and personalize the results of the automated assessment. Furthermore, each complexity factor was complemented with a potential optimisation measure to facilitate drug treatment (e.g. a patient leaflet). Complexity factors, key questions, and optimisation strategies were technically realized as tablet computer-based application, tested, and adapted iteratively until no further technical or content-related errors occurred.

**Results:**

In total, 61 complexity factors referring to the dosage form, the dosage scheme, additional instructions, the patient, the product, and the process were considered relevant for inclusion in the tool; 38 of them allowed for automated detection. In total, 52 complexity factors were complemented with at least one key question for preference assessment and at least one optimisation measure. These measures included 29 recommendations for action for the health care provider (e.g. to suggest a dosage aid), 27 training videos, 44 patient leaflets, and 5 algorithms to select and suggest alternative drugs.

**Conclusions:**

Both the set-up of an algorithm and its technical realisation as computer-based app was successful. The electronic tool covers a wide range of different factors that potentially increase the complexity of drug treatment. For the majority of factors, simple key questions could be phrased to include the patients’ perspective, and, even more important, for each complexity factor, specific measures to mitigate or reduce complexity could be defined.

## Background

Complexity of drug treatment arises from different characteristics in the medication regimen or external circumstances that may encumber drug administration for a patient. These complexity factors are manifold [1] and can potentially lead to nonadherence [2] and unplanned hospitalisations [3, 4]. They can be assigned to six categories depending on whether they are related to the dosage form (e.g. the use of transdermal patches [5]), the dosage scheme (e.g. an once weekly administration [6]), additional instructions concerning the use of a drug such as the coordination of the drug administration with a meal [7], the patient (e.g. swallowing difficulties [8]), the product (e.g. an intricate packaging [9]), or the medication process itself (e.g. frequently changing prescriptions [10]).

Besides consideration of single complexity factors such as the number of drugs in a medication regimen or the dosing frequency [11], the medication regimen complexity index (MRCI) is probably the most frequently used tool to assess complexity related to the medication regimen in a standardized way [12]. However, there is no tool currently available that covers all aspects potentially influencing complexity of drug treatment. Moreover, analysing and reducing the complexity of drug treatment remains time-consuming [13], even though the complexity in drug treatment could often be reduced significantly by simple measures [14]. In various health care settings, electronic tools have been developed to facilitate integration of decision aids in routine care [15] and also for reducing complexity in drug treatment it has been shown that individual complexity factors can be reduced by electronic decision aids [16]. Also the MRCI was transferred into an electronic decision aid [17] and tested in the home care setting [18]. Among patients in the intervention group, 8% of patients dropped below a MRCI score of 24.5 when the decision aid was used compared to 4.5% of patients when the decision aid was not used, suggesting that a tool that covers all areas of complexity would be even more successful.

However, there still remains the risk that patients do not adhere to the medication regimen after it has been changed, particularly if they have not been involved in the process of decision-making [19–21] and if complexity aspects relevant for the individual patient are not specifically addressed. Hence, a potential algorithm and its technical realisation should consider patient preferences to ensure that suggested changes and trainings are tailored to the patients’ needs with maximum chance of acceptance.

The aim of this study was therefore to develop an algorithm as well as its technical realisation that comprehensively assesses and reduces complexity of drug treatment. To this end, complexity factors must be specified that could be considered in such an automated analysis, appropriate strategies to assess and integrate the patients’ perspective must be developed and integrated, and, finally, feasible suggestions to mitigate complexity must be provided.

## Methods

As a prerequisite to the tool development, four key functionalities were defined:

Functionality I: Easy access to structured medication data.

Functionality II: Comprehensive, automated analysis of the drug treatment’s complexity based on distinct complexity factors.

Functionality III: Personalization of the automated analysis to the patient’s perspective and actual problems.

Functionality IV: Suggestion of appropriate measures to reduce or mitigate the identified complexity factors.

The respective functionalities were developed as follows:

### Access to structured medication data

In Germany, all patients with chronic medication intake are entitled to a nationally standardized medication schedule providing information on the active ingredient, the brand name, the dosage form, the strength, the dosing frequency, the unit, instructions for use, and the indication of each drug [22]. This paper-based document has a two-dimensional data matrix code which can be scanned to transfer the medication data and, thus, offers an easy access to structured prescription data (XML-data). After starting the tool, the user should be able to scan the data matrix code of medication schedules to upload the medication data for analysis.

### Automated assessment of complexity factors

Based on all available information of a medication schedule, the tool should comprehensively assess complexity factors that actually represent a relevant problem for patients in routine care and, thus, should be considered in the automated analysis. To do so, we combined a literature-based with a qualitative approach. As described previously by our group, 91 complexity factors were identified [1] which had to be rated for their relevance by experts to decide upon their inclusion in the tool. We therefore formed an expert panel of 10 experts (clinical pharmacologists, general practitioners, and pharmacists involved in the development of the electronic tool and their colleagues) to rate factors previously characterised in the literature as having little relevance (1 point), medium relevance (2 points), and high relevance (3 points). Complexity factors rated with less than 25 points were subsequently discussed within the expert panel to decide whether or not they should be further considered in the development process of the tool. If during the expert discussion new complexity factors were identified, these factors could be included in the final list if all experts agreed.

The adapted set of complexity factors was then rated in a second round by another six experts to verify the result. The final decision on the factors that should be considered in the automated assessment was made by the authors based on the results of the expert ratings and the influence of each complexity factor on complexity of drug treatment. For example, some complexity factors only indirectly influence complexity and therefore could not or only with difficulty be optimised within routine care (e.g. a lack of interest in drug treatment or a low income). For the final set of complexity factors, feasibility of automated detection was checked and respective rules and criteria for automated detection were specified whenever possible.

### Personalization of the analysis to patient needs

The patients’ perspective on the identified complexity factors and, thus, the relevance for the individual patient should be assessed interactively and stored in the tool. To do so, we allocated so called key questions to all factors that could be automatically detected by the tool. For example if a patient had to use an inhaler, a key question addressing problems with correct inhaling was phrased. These key questions were developed following a previously described approach [23]. Briefly, it is a five-step process involving patients and health care professionals to develop and validate key questions. Thereby it can be ensured that the key questions are specific to a complexity factor, comprehensible for patients, suitable to indeed identify a patients difficulties and implementable in patient visits.

For factors that could not be detected automatically (e.g. cognitive impairment), distinct questions should be phrased to assess whether this factor was relevant for the patient. Consequently these distinct questions cannot be linked to the automated detection of complexity factors but allow to consider additional factors besides the automated analysis when reducing complexity of drug treatment with the tool. As an example, swallowing problems cannot be detected from a medication schedule, but were identified as a relevant complexity factor.

### Suggestion of optimisation measures tailored to the patient’s needs

Based on the patients’ responses to key questions, optimisation measures should be proposed selectively for factors that are relevant for the patient. Hence, at least one potential strategy to reduce or mitigate the respective complexity factor was allocated to each factor and a key question or a distinct question for its detection was phrased. The strategies belonged to one of the following three categories:

- Recommendations for action of the health care provider, e.g. to recommend a dosage aid
- Training material, e.g. patient leaflets
- Algorithms to modify the medication regimen, e.g. by selecting an alternative drug with less frequent dosing

### Technical implementation of the functionalities

The functionalities were then implemented in a windows-based computer app using the programming language C Sharp (.NET 4.5) and the GUI toolkit Windows Forms. This app can access current medication data on all drugs available in Germany in a Microsoft SQL database, meaning that the XML-data obtained from the medication schedule can be compared with this database to identify matching entries. On the medication level this was done via the so-called “Pharmazentralnummer” (Pharmacy Product Number) which is an ID unique to each medicinal product in Germany. Consequently, additional information about a medication (e.g. the type of inhaler) can be retrieved, which is important for the targeted proposal of key questions and optimisation measures. Moreover, appropriate alternative drugs can be suggested by the algorithms as an optimisation measure in this way.

For some complexity factors, identification is based on key words in the free texts of the medication schedule. To this end, respective keywords were defined, allowing the XML-Data of the medication schedule to be screened for them.

To ensure the reliable and error-free performance, the tool was tested with exemplary medication schedules to find potential technical or content-related errors. Therefore fictional medication schedules were created to ensure that automated detection of each complexity factor could be iteratively checked. Thereby, the user interface design was optimised for the intended use by ensuring a sufficient font size, a clear presentation, and the ease of use. Testing was independently and iteratively performed by two pharmacists.

## Results

### Access to structured medication information

**Fig. 1:**
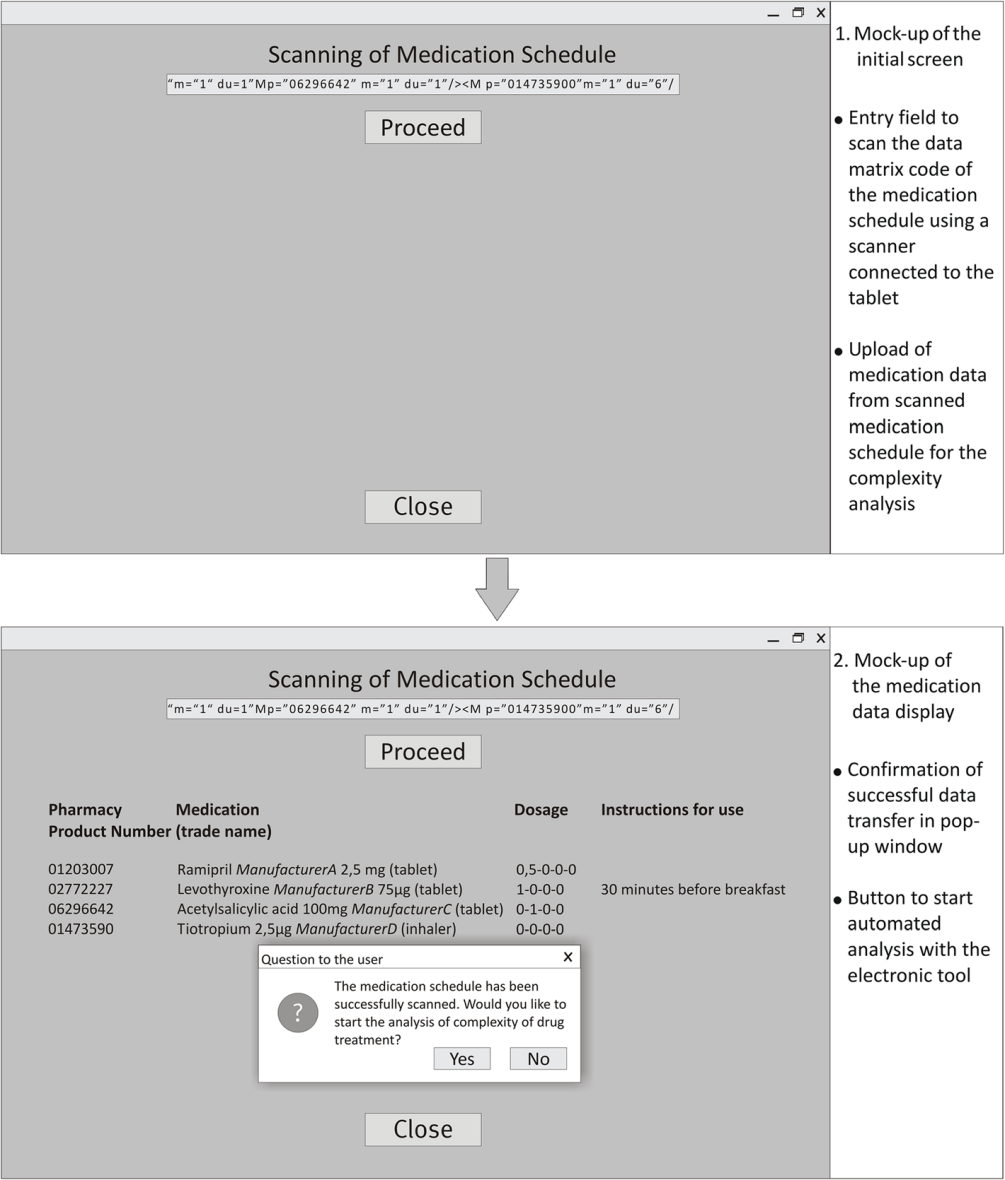
Technical realisation of functionality I

The tool was made available on tablet computers. Via a specific scanner, the data matrix code of the medication schedules could be scanned (Fig. 1).

### Automated assessment of complexity factors

Out of the 91 factors increasing treatment complexity [1] 34 complexity factors were excluded due to a low relevance according to the expert panel. Four complexity factors were additionally included based on expert opinion, i.e. diverse storage conditions of the patient’s individual drugs; administration of only one drug at one specific point in time, as a challenging dosage scheme; the same active ingredient in different preparations, which might potentially confuse the patient; and occasional, episodic treatment with a drug, e.g. an antibiotic.

Accordingly, the final list of complexity factors comprised a total of 61 factors (Table 1), which where almost evenly distributed amongst all six categories [1]: 13 factors were assigned to the category dosage form, 14 to the category dosage scheme, eight to the category additional instructions, ten to the category patient, five to the category product, and 11 to the category process.

**Table 1:** Final set of complexity factors and the allocated key questions and optimisation measures

Complexity factor	Identifiable in medication schedule?	Characteristic in medication schedule	Key question	Optimisation measure
**Dosage forms**				
Inhalers				
Metered dose inhaler	yes	Pharmacy Product Number	Many patients find it difficult to pull the trigger of their inhaler and to inhale at the same time. Are you having any trouble using your inhaler? For how many seconds after inhalation do you hold your breath?	Recommendation of action Training material
Elpenhaler			Do you always manage to insert and remove the blister strip without any problems? For how many seconds after inhalation do you hold your breath?	Training material
Nebulisers			Many patients find it difficult to measure the exact number of drops. Do you have any problems measuring the dose for your nebuliser?	Training material
Capsule-based inhalers			For how many seconds after inhalation do you hold your breath? Do the capsules contain powder residues after inhalation	Training material
Other inhalers			For how many seconds after inhalation do you hold your breath?	Training material
Injection devices (non-prefilled)	yes	Pharmacy Product Number	How frequently do you change the injection site?	Algorithm
Injection devices (prefilled)	yes	Pharmacy Product Number	How frequently do you change the injection site?	Training material
Transdermal patches	yes	Pharmacy Product Number	Do you sometimes have to change your patch more often than prescribed, e.g. because it does not last or the effect wears off too quickly?	Training material
Nasal preparations (prescription-only)	yes	Pharmacy Product Number	Many patients have the feeling that they have to use more spray/drops than prescribed in order to achieve a sufficient effect. Does this reflect your experience?	Training material
Solid dosage forms for oropharyngeal use	yes	Pharmacy Product Number	This drug should not be swallowed but should be applied to the oral cavity. Have you ever swallowed this drug by mistake?	Recommendation of action
Liquid dosage forms for oropharyngeal use	yes	Pharmacy Product Number	This drug is not to be swallowed. It should be applied to the oral cavity. Have you ever swallowed this drug by mistake?	Recommendation of action
Ophthalmic preparations				
Drops	yes	Pharmacy Product Number	Do you always succeed in inserting a drop into the conjunctival sac at the first attempt? Do you always keep both eyes closed after the drop?	Training material
Ointment/ creme/ gel			Do you always succeed in inserting the correct amount of this medication into the conjunctival sac at the first attempt?	Training material
Rectal preparations	yes	Pharmacy Product Number	Many patients have problems with the use of this drug. Does this equally apply to you?	Training material
Dermatological preparations (prescription-only)	yes	Pharmacy Product Number	Many patients find it difficult to choose the right amount of cream or ointment. Do you equally have problems using your cream or ointment?	Recommendation of action
Liquid oral dosage forms				
With measuring device	yes	Pharmacy Product Number	Many patients describe the measurement of a liquid drug as difficult. Do you have any difficulties with the measurement - for example when using the enclosed dosage device?	Recommendation of action Training material
Dry syrup			Many patients report problems with the preparation of their liquids, e.g. because a strong foam develops. Do you have any difficulties with the preparation?	Training material
Drops			Counting drops is difficult for many patients.Do you have any difficulties concerning this?	Training material
Otological preparations	yes	Pharmacy Product Number	Patients often find it often difficult to use ear drops - for example using the dropper with a tilted head. Does the application also cause problems for you?	Training material
Vaginal preparations	yes	Pharmacy Product Number	Many patients are insecure about the vaginal application of drugs. Does this also apply to you?	Training material
**Dosage schemes**				
Once weekly administration	Yes	Free text (definition of keywords)	This drug should be used once a week. Is it difficult for you to use this drug always at the same day of the week?	Recommendation of action
Tablet splitting	Yes	Dosage scheme	Do you find it difficult to split your tablets consistently into pieces that have the same size?	Algorithm Training material
Total number of drugs	Yes	Lines in medication schedule	When taking a multitude of drugs simultaneously, many patients feel overburdened. Do you have difficulties keeping track of your drugs?	Algorithm Recommendation of action
Administration more than two times daily	yes	Dosage scheme	Is it a problem for you to take your medication several times a day in everyday life?	Recommendation of action
Administration at lunch time	Yes	Dosage scheme	According to your medication schedule, you should use this drug at noon. Many patients find it difficult to actually do this in everyday life. Have you found it difficult to take your drugs at noon?	Recommendation of action
Administration every two days or less frequently	Yes	Free text (definition of keywords)	Is it difficult for you to remember taking this drug because it is not used every day?	Recommendation of action
Fixed dosing interval	Yes	Free text (definition of keywords)	Are you able to keep the exact intervals between the administrations of this drug in everyday life?	Recommendation of action
Use of multiple doses concurrently	Yes	Dosage scheme	This drug should be used more than once at the same point in time. Do you find it difficult to use this drug repeatedly each single time?	Algorithm
Different doses of the same active ingredient at different times of day	Yes	Dosage scheme	You use different doses of this drug during one day. Do you sometimes accidentally mix up these doses?	Recommendation of action
Variable dosing	Yes	Dosage scheme	No exact dose is indicated for this drug. Does this make you insecure about how to take this drug?	Recommendation of action
Occasional, episodic drug treatment	Yes	Free text (definition of keywords)	This drug is used for a limited time only. Are you able to integrate this drug into your daily routine?	Training material
Only one drug at one specific point in time	Yes	Dosage scheme	Your medication schedule specifies that you use this drug at a separate time. Do you ever forget to take this drug?	Recommendation of action
Pro re nata (as needed) medication	Yes	Free text (definition of keywords)	In your medication schedule, it is specified that you may use this medication if necessary. Do you know the medical condition that is treated with this drug? Do you know what dose you can use?	Recommendation of action
The same active ingredient in different preparations	Yes	Pharmacy Product Number	These two drugs contain the same active ingredient. Is there a risk of you confounding these drugs?	Recommendation of action
**Additional instructions**				
Meal-dependent administration	Yes	Free text (definition of keywords)	Do you find it difficult to coordinate the daily use of your medication with your meals?	Recommendation of action
Crushing tablets	Yes	Free text (definition of keywords)	This drug is to be crushed. Do you have any difficulties crushing this drug in such a way that it is it easier to take?	Algorithm
Disintegrating tablets, capsules and powders	Yes	Free text (definition of keywords)	This drug should be dissolved before use. Is your drug always completely dissolved?	Recommendation of action
Administration at fixed times of the day	Yes	Free text (definition of keywords)	Your medication schedule specifies that this drug should be used at a certain time of the day. Are you able to integrate this into your daily routine?	Recommendation of action
Intake with advised liquid (or food)	Yes	Free text (definition of keywords)	Do you find it difficult in everyday life to remember taking this medication only with the special liquid or food?	Recommendation of action
Opening capsules	Yes	Free text (definition of keywords)	These capsules are to be opened before use. Do you have any difficulties opening the capsules?	Training material
Increasing doses	Yes	Free text (definition of keywords)	Can you tell me in your own words how you should increase the dose?	Recommendation of action Training material
Decreasing doses	Yes	Free text (definition of keywords)	Can you tell me in your own words how you should reduce the dose?	Recommendation of action Training material
**Patient characteristics**				
Cognitive impairment ^a^	No	–	Do you find it difficult to remember names, times, or dosages?	Recommendation of action
Physical impairment ^a^	No	–	Many patients do not manage to use their drugs without experiencing some problems. For example, they may lack strength or may no longer be able to read instructions. Do you also have physical restrictions when it comes to the use of your drugs?	Recommendation of action
Low health literacy	No	Not considered in tool		
Lack of knowledge regarding disease/drug treatment	No	Not considered in tool		
No support in drug handling	No	Not considered in tool		
Busy lifestyle	No	Not considered in tool		
Poor numeracy skills	No	Not considered in tool		
Swallowing difficulties	No	–	Do you have problems swallowing your drugs?	Algorithm Recommendation of action Training material
Use of alternative medicines	No	Not considered in tool		
Alcohol or illicit drug use	No	Not considered in tool		
**Product characteristics**				
Similar drug names ^a^	No	–	Do you have problems distinguishing your drugs because they look similar or their names sound alike?	Recommendation of action
Similar drug appearance ^a^	No	–		Recommendation of action
Patient-unfriendly nature of solid oral dosage forms	No	Not considered in tool		
Patient-unfriendly nature of liquid oral dosage forms	Yes	Pharmacy Product Number	Have you ever not taken your drug because the smell, taste, or consistency disturbed you?	Recommendation of action
Intricate packaging ^a^	No	–	Many patients find it difficult to remove their drugs from the packaging. Do you have any difficulties with the packaging of one of your drugs?	Recommendation of action
**Process characteristics**				
Lack of training in dosage form use	Yes	Pharmacy Product Number	Have you been advised on how to use this drug? Do you think that an explanation of how to use your drug would make it easier for you to perform your therapy?	Training material
Frequently changing prescriptions ^a^	No	–	Have your drugs changed recently, for example because a drug is from another brand or because a new drug has been prescribed? Do you find these changes difficult?	Recommendation of action
Changes in existing medication regimen ^a^	No	–		
New prescription ^a^	No	–		
Frequent generic substitution ^a^	No	–		
Changes in tablet color or shape ^a^	No	–		
Hospital discharge ^a^	No	–		
Lack of comprehensibility and transparency of the instructions for drug treatment	No	Not considered in tool		
Complex measurements (self-performed)	Yes	Pharmacy Product Number & Free text (definition of keywords)	Do you feel safe adjusting your dose after measuring blood glucose/ blood coagulation?	Recommendation of action
No use of medication schedule ^a^	No	–	Do you use your medication schedule in everyday life, e.g. when you administer or prepare your drugs?	Recommendation of action
Diverse storage conditions ^a^	No	–	Do you store all of your drugs in one place? Has it ever happened that you forget to take drugs that you do not keep with others?	Recommendation of action

^a^complexity factors considered in a distinct question

Of 61 complexity factors, 38 (62%) could be identified automatically from the structured medication data of a medication schedule, either by using structured information, such as the dosage scheme, or by searching index words in free-text fields. For instance, for the complexity factor “Meal-dependent administration”, the detection was based on the keywords “meal”, “food”, “eat”, “breakfast”, “lunch”, or “dinner”.

### Personalization of analysis to patients’ needs

For every complexity factor that allowed for automated detection, at least one specific key question was developed (Table 1 [23];). For three dosage forms different devices are available that differ substantially in their way of use. Therefore, it was necessary to develop different key questions for the different devices.

For 14 of the remaining 23 complexity factors, which could not be identified automatically, eight distinct questions were developed and implemented in the software that allowed for detection of the respective factors (e.g. swallowing difficulties). Hence, a total of 52 complexity factors could ultimately be considered in the analysis of treatment complexity by the electronic tool. The remaining nine complexity factors were too vague to be included as single complexity factors. However, we phrased an open-ended question inviting the patients to report any further difficulties with their drug treatment that have not been addressed by the previously identified complexity factors (i.e. “Is there anything else that is difficult for you in using your medication?”).

### Recommendation of optimisation measures tailored to the patients’ needs

A total of 105 optimisation measures to reduce or mitigate treatment complexity were newly developed or adapted for inclusion in the tool (Table 1).

These included five algorithms suggesting alternative treatments (e.g. different dosage forms or drugs with different dose strengths), 44 patient leaflets (e.g. illustrating how to use a particular dosage form), which were developed based on already existing educational material [24–26], 27 training videos from the Deutsche Atemwegsliga e.V. (German Airway League, a registered, charitable association informs patients and doctors about respiratory and lung diseases and is not sponsored by the pharmaceutical industry) for inhaler devices [25] and, 29 recommendations for action referring to three topics:

I) Recommendations of aids that facilitate medication administration and therefore could be proposed to the patient, for instance a spacer.
II) Recommendations to explain a certain aspect of medication administration to the patient, for example the administration of dosage forms for oropharyngeal use.
III) Recommendations to review a certain aspect of the medication regimen and to consider changing the medication regimen, for example to change the time of administration.

### Technical implementation of the functionalities

**Fig. 2:**
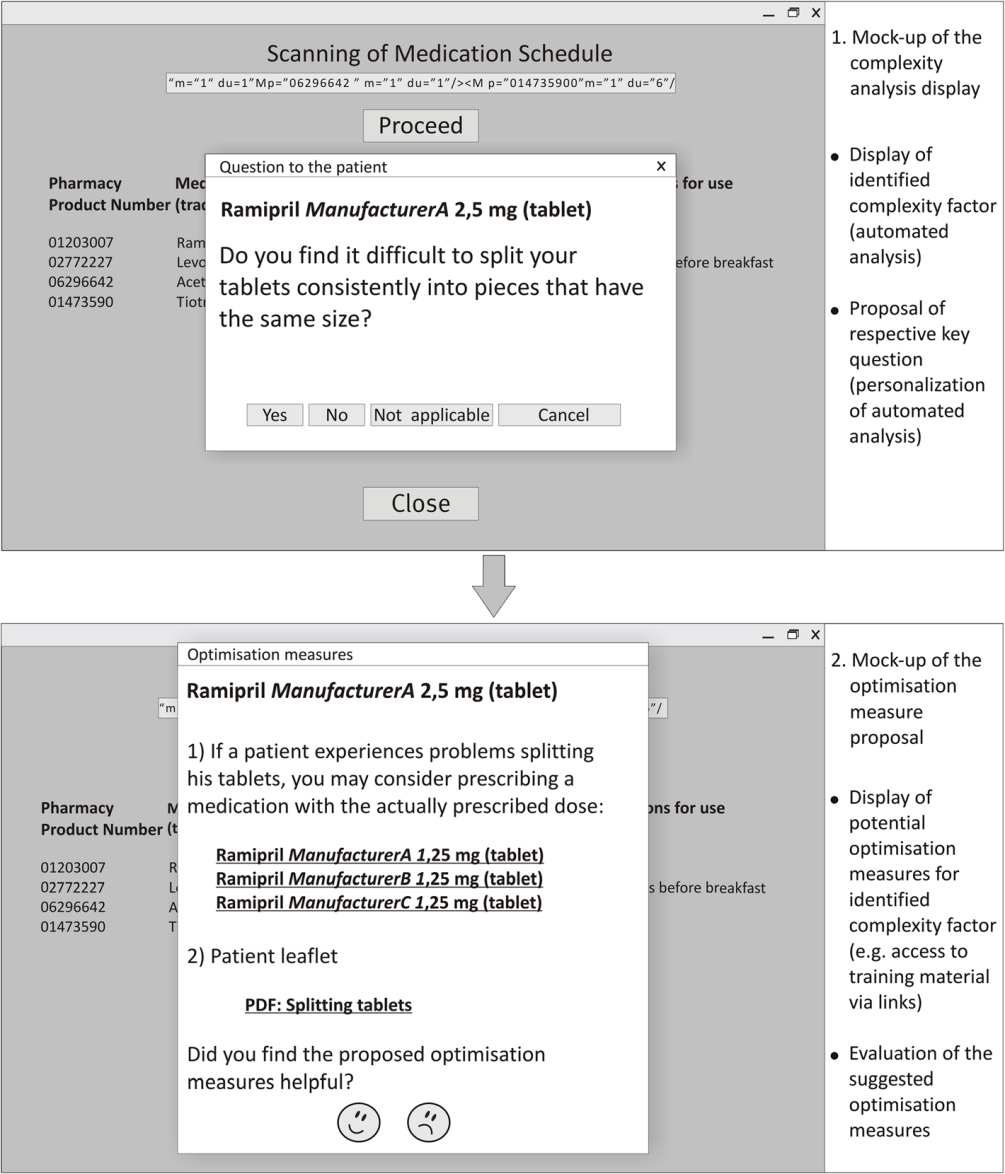
Technical realisation of functionalities II-IV

**Fig. 3:**
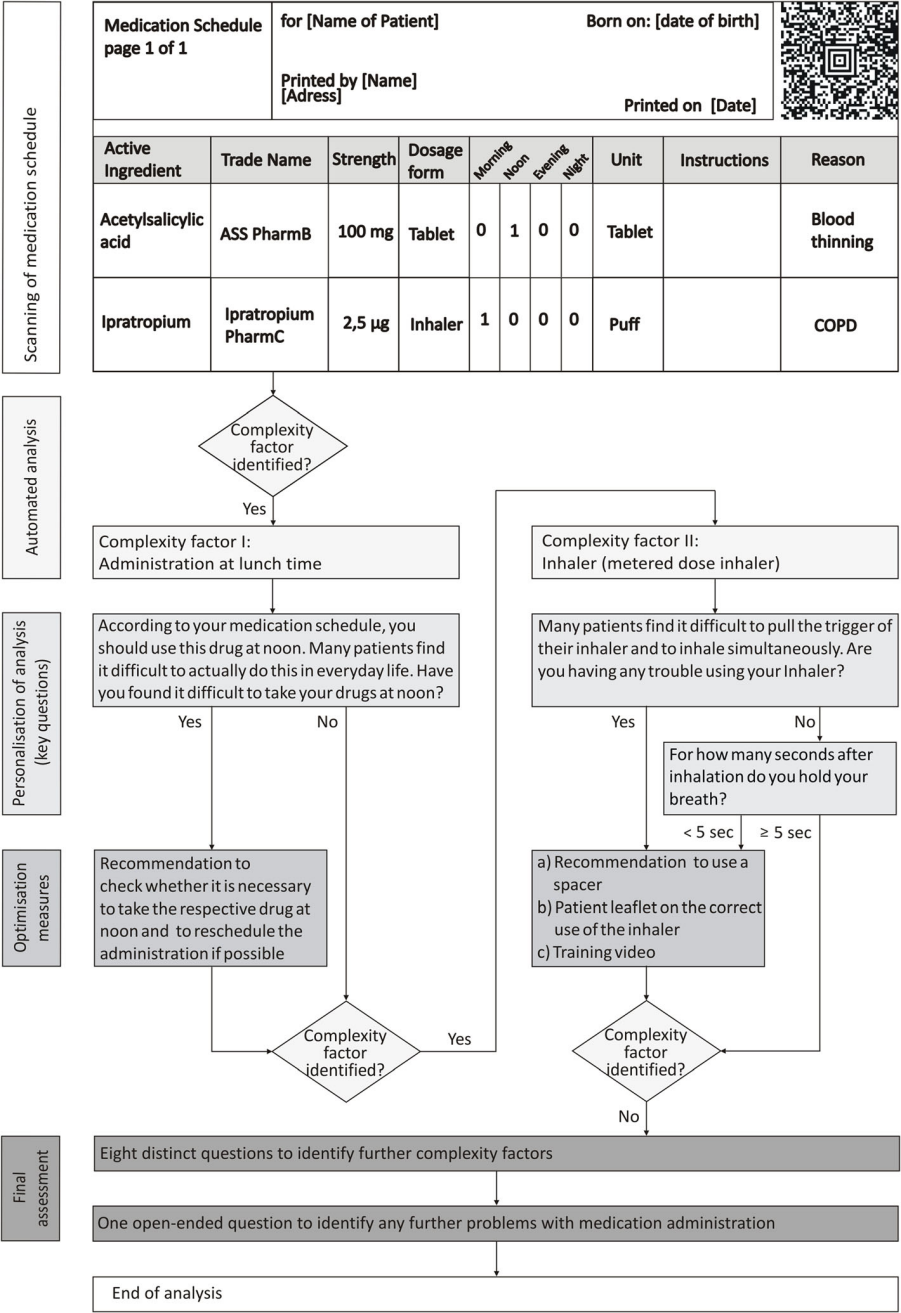
Exemplary workflow to analyse and reduce complexity of drug treatment using the electronic tool

The four functionalities were integrated and aligned in the tool (Fig. 2) in a way that the user is automatically guided through the application (Fig. 3). The tool was tested with approximately 50 exemplary medication schedules until no further technical or content-related errors occurred. Testing revealed that the tool reliably identifies complexity factors and, thus, proposes targeted optimisation measures to reduce complexity in drug treatment.

## Discussion

The developed electronic tool comprised 52 complexity factors covering a wide range of factors contributing to treatment complexity. Thereby, almost two in three complexity factors could be automatically detected if structured medication data was entered as provided by a medication schedule. This applied in particular to the complexity factors related to the dosage form, the dosage scheme, or additional instructions, thereby confirming the results of the MRCI decision aid [12, 17].

In contrast, most complexity factors relating to the patient, the product, or the medication process could not be detected by the electronic tool, either because this information could not be deduced from data currently stored on the medication schedules or structured information to develop respective algorithms is currently not available. Hence, to integrate also these factors, either alternative data sources such as electronic health records would be needed (e.g. to have access to the patients’ diseases) and more thorough information particularly on product characteristics would be required, e.g. on the packaging of each drug or individual tablet shape and size. With such information, the medication schedule could for instance be searched for packages that are difficult to open such as child-resistant containers [9] and algorithm-based packages that are easier to open could be suggested.

Moreover, to develop more complex algorithms, e.g. for the automated detection of sound-alike drugs, either structured data on resembling drug names could be integrated or an algorithm to identify such drug pairs based on linguistic approaches, such as an analysis of orthographic and phonetic similarities of all drug names in a medication regimen, could be specified [27].

In total, about one third of the complexity factors considered relevant by the experts could not be automatically evaluated by our complexity tool. To compensate this shortcoming in the short term, we used distinct questions that complemented the automated assessment and address potential problems of the patient with the current treatment regimen. This approach proved to be sufficient for many relevant challenges in a patient’s life such as swallowing difficulties, diverse storage conditions, or complicated packaging. However, factors relating to the patients’ capabilities, their health literacy or their life style could not be assessed by a single question and hence were not considered in our assessment even though they were rated as important by the experts in the first place.

Nevertheless, the inclusion of health care professionals in the selection of the complexity factors considered in the tool and the development of the key questions ensures that the final electronic tool meets the needs of routine care. Thus, it should be made sure that the tool can be implemented easily in patient care and that relevant patient problems are addressed, which can be solved or mitigated directly by a health care professional. However, in order to reduce the influence of the professional background and the personal experiences in patient care of each individual on the final algorithm, several health care professionals with different professions or specialisations have been involved. In general, the included key questions clearly distinguish the analysis with our electronic tool from a simple complexity assessment and allow for patient-specific selection of optimisation measures (personalized intervention). The actual impact of this approach on routine care will be tested in a prospective pilot study and compared to other approaches to reduce treatment complexity that do not consider the patient preferences. A future widespread use of the tool in the health care system is conceivable because it could be used in different settings, such as pharmacies, ambulatory care and, also hospitals. Even individualization of the recommended optimisation measures to the setting appears possible; for use in a pharmacy, an algorithm to change the dosage form could for example be replaced by the recommendation for action to contact a doctor and suggest an alternative drug. Furthermore, specific patient populations might need adapted optimisation measures, such as blind or visually handicapped patients who will benefit less from standard information brochures but might need materials with verbal explanations. This work has several limitations. First, the analysis of complexity of drug treatment is based on structured medication data from the nationally standardized medication schedule and, thus, there is a risk that not all of the patient’s drugs are considered, e.g. because the medication schedule is not up-to-date or not comprehensive. In particular, pro re nata (as needed) medication and non-prescription drugs might not be included in the medication schedule, for example because they are forgotten or not considered as relevant by health care professionals and patients. However, in order to comprehensively assess and reduce treatment complexity with the electronic tool, it would be essential to include all medications used by the patient in the medication schedule.

Second, the automated identification of complexity factors is partly based on the identification of index words in the free text fields of the medication schedule. Therefore, accurate identification depends on the correctness and comprehensiveness of the medication schedule and correct spelling of the index words. Moreover the definition of index words might not be comprehensive enough to cover all possible notations health care professionals might use. In particular, the use of non-standard abbreviations in the free text fields of the medication schedule could result in complexity factors not being detected in the automated analysis. Third, the tool was tested with a limited number of exemplary medication schedules. It is, therefore, possible that not all possible cases are considered when specifying criteria for the automated detection of individual factors; hence, some complexity factors may have escaped detection by the electronic tool. However, the tool will be tested in a pilot study with a large number of actual medication schedules issued by different prescribers allowing further optimisation of the electronic tool and adaptation to routine care.

## Conclusions

We developed an algorithm-based electronic tool that covered a wide range of different factors that are known to increase complexity of drug treatment. The majority of factors could be identified in an automated analysis by the tool and key questions could be phrased to assess whether these complexity factors indeed pose a problem for a specific patient. Relevant complexity factors that were not suitable for an automated detection were considered by distinct questions and a general, open-ended question. The electronic tool combines an automated screening with a personalized intervention to comprehensively assess, mitigate, or antagonize treatment complexity. This electronic tool is designed to tailor medication regimens to the needs of the individual patient and is now ready for testing in a prospective pilot study.

## Data Availability

Data sharing is not applicable to this article as no reusable datasets were generated or analyzed during the current study.

## Abbreviation

MRCI: Medication Regimen Complexity Index
